# HADF-Crowd: A Hierarchical Attention-Based Dense Feature Extraction Network for Single-Image Crowd Counting

**DOI:** 10.3390/s21103483

**Published:** 2021-05-17

**Authors:** Naveed Ilyas, Boreom Lee, Kiseon Kim

**Affiliations:** 1 Department of Biomedical Science and Engineering, Gwangju Institute of Science and Technology (GIST), Gwangju 61005, Korea; naveedilyaas@gmail.com; 2 School of Electrical Engineering and Computer Science, Gwangju Institute of Science and Technology (GIST), Gwangju 61005, Korea; kskim@gist.ac.kr

**Keywords:** deep learning, CNNs, crowd analysis, crowd counting

## Abstract

Crowd counting is a challenging task due to large perspective, density, and scale variations. CNN-based crowd counting techniques have achieved significant performance in sparse to dense environments. However, crowd counting in high perspective-varying scenes (images) is getting harder due to different density levels occupied by the same number of pixels. In this way large variations for objects in the same spatial area make it difficult to count accurately. Further, existing CNN-based crowd counting methods are used to extract rich deep features; however, these features are used locally and disseminated while propagating through intermediate layers. This results in high counting errors, especially in dense and high perspective-variation scenes. Further, class-specific responses along channel dimensions are underestimated. To address these above mentioned issues, we therefore propose a CNN-based dense feature extraction network for accurate crowd counting. Our proposed model comprises three main modules: (1) backbone network, (2) dense feature extraction modules (DFEMs), and (3) channel attention module (CAM). The backbone network is used to obtain general features with strong transfer learning ability. The DFEM is composed of multiple sub-modules called dense stacked convolution modules (DSCMs), densely connected with each other. In this way features extracted from lower and middle-lower layers are propagated to higher layers through dense connections. In addition, combinations of task independent general features obtained by the former modules and task-specific features obtained by later ones are incorporated to obtain high counting accuracy in large perspective-varying scenes. Further, to exploit the class-specific response between background and foreground, CAM is incorporated at the end to obtain high-level features along channel dimensions for better counting accuracy. Moreover, we have evaluated the proposed method on three well known datasets: Shanghaitech (Part-A), Shanghaitech (Part-B), and Venice. The performance of the proposed technique justifies its relative effectiveness in terms of selected performance compared to state-of-the-art techniques.

## 1. Introduction

A crowd can be defined as a complex phenomenon due to constant interaction among people within the crowd, distinct behaviors of individuals, and inter-object occlusions. The estimated population will reach 10 billion in 2056 (United Nations prediction) [1]. Due to the rapidly increasing population, the number of cities will also increase, leading to more crowd-like activities such as sports, religious gatherings, cultural festivals, public transport terminals, and concerts. With limited space and resources in these mega-gatherings, there is a need to enhance security and safety arrangements. In this way understanding crowd dynamics is a challenging task. Understanding of crowd behavior involves multidisciplinary research such as urban planning, civil engineering, and studying and analyzing crowd behavior, especially in abnormal situations. Similarly, organizers of mega-gatherings like marathons, political protests, and concerts perform crowd analysis to plan evacuation from any unusual events.

The density of people in a specific area and their spatial distribution are two main indicators to understand any type of crowded scene. In the recent past, a number of convolutional neural network (CNN)-based algorithms have been proposed to mitigate the difficulties of crowd counting [2]. CNNs have significant ability to learn deeper and powerful features. Existing CNN-based crowd counting (CC) techniques enhance the counting accuracy by using well-known networks such as multi-column, multi-tasking, dilated, and de-convolutional [3] networks. These networks have been widely used individually or in combination with each other to increase the performance at the cost of major shortcomings, such as large amounts of training time, ineffective branch structure, sparse pixel sampling rates, information loss, and extraction of irrelevant information. The authors of [4,5] used a multi-column architecture for density estimation by taking advantage of different receptive fields. The same size of kernels in each column results in extraction of a specific set of density scenes. In addition, the authors of [6] proved through detailed experiment that multiple parallel columns [4,5] extract similar types of features irrespective of different kernel sizes.

Based on these observations, we propose an attention-based dense feature extraction network for single-image crowd counting (HADF-Crowd) method. Our model comprises three main modules: (i) backbone network, (ii) dense feature extraction module (DFEM), and (iii) channel attention module (CAM). A backbone network with strong transfer learning capability is used to obtain the simple to complex features. The DFEM consists of four sub-modules called dense stacked convolution modules (DSCMs), which are densely connected with each other to enable feature sharing from lower layers to higher layers. The CAM is capable of exploiting the class-specific response to obtain rich feature representation in the final layers.

The main contributions of our research are summarized as follows.

- We design a deeper and denser attention-based CNN-based CC network to obtain abrupt to continuously varying scale features. Densely organized DSCMs extract and aggregate the local to global information in a final density map.
- The proposed network, composed of a backbone, DFEM, and CM, enhances the ability of the network to obtain general, contextual, and perspective-varying features for better CC accuracy.
- The rich semantic feature representation in the final layers is obtained by modeling the dependency among channels, thus combining the low-to-high semantic features for enhanced counting accuracy.
- The proposed approach is responsible for aggregating task-independent and task-specific features at higher layers from lower and middle-lower layers, enhancing the estimation accuracy.

## 2. Related Work

Due to the boom in CNN-based CC techniques, many CC techniques have used CNNs to enhance the counting accuracy. These approaches usually focus on typical techniques such as multi-scale [7,8,9], context [10,11,12], and multi-task [13,14,15,16]. Recently, researchers have dived deep into CNN-CC techniques to handle scale variation issues. For instance, the authors of [4] presented a multi-column network that was used to combine the features from multiple branches, thus combining the multiple receptive fields. Whereas, in Switch-CNN [5], three regressors with a multi-column approach are used. Each of the regressors is trained on different density levels. The input image is directed to one of the three regressors based on the density distribution in the input image patch. Further, the authors of [17] incorporated a multi-column network with local and global context-aware modules to enhance the CC accuracy. SaNet [18] uses a scale aggregation network to handle the perspective distortion. To reduce the complexity of the network and enhance the quality of the density map, the authors of [6] proposed a single-column dilated network with smaller and the same sizes of filters. Later, the authors of [19] proposed an attention-aware network for CC. The model is composed of an encoder–decoder framework and a conditional random field (CRF) with an attention module. The CRF and attention module are incorporated in the encoder–decoder framework for better CC accuracy. The authors of [20] presented a density-aware network (DAN) for CC, comprising a density-aware network, an enhancement module, and a feature fusion network. The DAN moves further into sub-networks that are pre-trained on multiple density levels.

The authors of [21] presented a CC network that employs a VGG-16 as a backbone network with a residual learning method to obtain a fine density map. The residual learning is guided by a confidence weighting mechanism by only allowing the flow of high-confidence residuals. The authors of [22] proposed a fusion technique mainly comprising two modules. A multi-level bottom–top and top–bottom fusion method (MBTTBF) was mainly used to aggregate features from shallower to deeper layers and vice versa. In addition, scale complementary feature extraction blocks (SCFBs) enable the flow of important information along the fusion path. An encoder–decoder-based CC approach was proposed by authors of [23]. It has multiple decoding paths to aggregate the features at different stages. Further, skip-connections are used to obtain multi-scale features. The authors of [24] proposed a CC network to learn complementary scale information by fusing the multi-scale features. The network performs the whole RGB image-based inference to facilitate model learning and decrease contextual information loss. The whole network is trained in an end-to-end manner with the naive Euclidean loss.

## 3. The Proposed Approach

**Figure 1:**
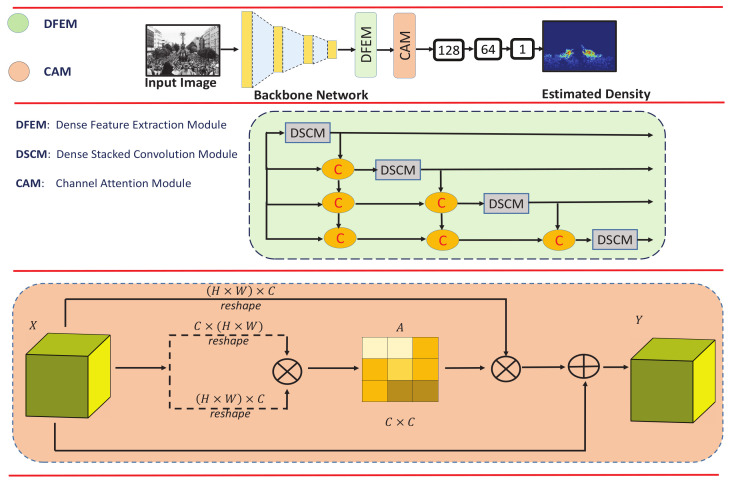
The overview of HADF-Crowd, a hierarchical attention-based dense feature extraction network for single-image crowd counting (**top**). The dense feature extraction module (DFEM) with four deep DSCMs densely connected with each other (**middle**). The channel attention module (CAM) (**bottom**).

The architecture of the proposed approach is shown in Figure 1. Firstly, HADF-Crowd counting starts from ground truth density estimation. Secondly, our proposed model employs a backbone network (inspired from VGG-16), which is used to obtain simple to complex deep features. Thirdly, our proposed network utilizes DFEMs, which enable the network to extract deep and relevant features. Fourthly, multiple DSCMs within a DFEM are densely connected with each other, thus enhancing the ability of the network to handle perspective distortion while propagating the information to higher layers. Thus, the output of one DSCM has direct access to each layer of the subsequent DSCMs, resulting in contiguous information passing to high-level layers. Finally, the CAM with strong modeling of class-specific responses uses the aggregated information from lower layers to obtain rich semantic features.

### 3.1. Backbone Network

**Figure 2:**
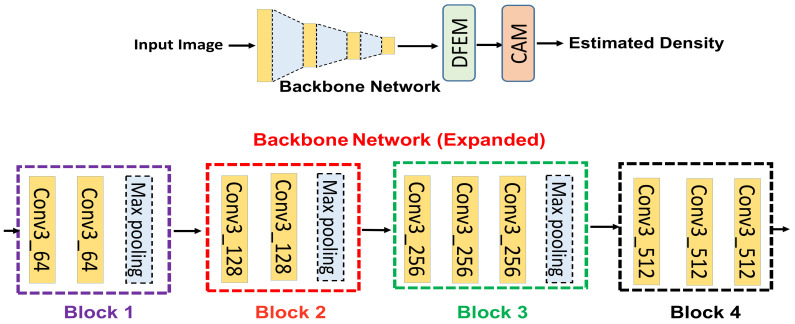
The Overview of HADF-Crowd (**top**). The detailed architecture of the backbone network (**bottom**). It is a single-column network with four blocks starting from block 1 and ending with block 4.

The lack of training data is one of the major hurdles for training a deep neural network (DNN). Similarly, CNN-based CC faces different types of challenges, like small amounts of training data, perspective distortion, and variation of density levels within a specific scene. To mitigate these above mentioned challenges, transfer learning can be employed to learn features efficiently, save the training time, and increase the performance of the DNN without increasing the computational resources. As crowd counting belongs to regression-based learning, however, most of the existing DNNs are trained for logistic regression instead of regression-based tasks. However, this problem has been resolved by the authors of [25] by revealing the nature of feature learning by the front- and back-end of the DNN. They revealed that the front-end of the network learns task-independent general features, whereas the back-end of the network learns task-specified features. Hence, based on these considerations, we used a pre-trained VGG-16 [26] network as our backbone network to obtain the maximum advantages as discussed above. The backbone network comprises different types of layers such as convolution, ReLU, and max pooling. We used only ten layers from VGG-16 to reduce the computational cost. In this way, it is a flexible architecture to concatenate with DFEM for density estimation. The detailed architecture of the backbone network is shown in Figure 2. The backbone network consists of four blocks (block 1, block 2, block 3, block 4). The four blocks have same filter size with different numbers of channels as shown in Figure 2 (bottom). Block 1 is responsible for obtaining the general features like lines, edges, contours, etc. The rest of blocks are useful for obtaining the more detailed features. In addition, the backbone network has strong transfer learning ability, which further enhances the overall performance of the proposed algorithm.

### 3.2. Dense Feature Extraction Module (DFEM)

**Figure 3:**
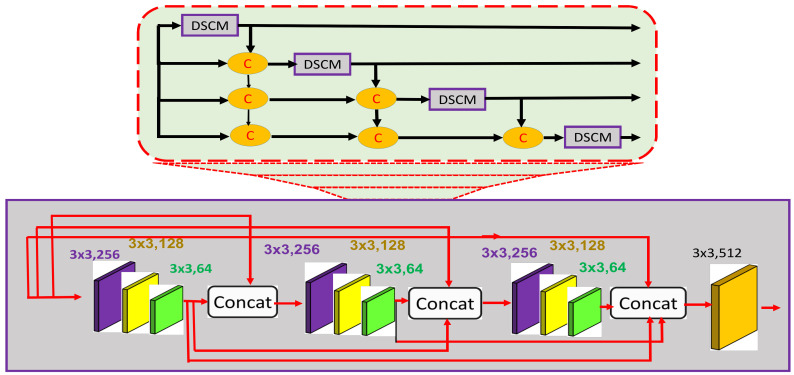
The expansion of DFEM with multiple DSCMs densely connected with each other (**top**). The internal architecture of each of DSCM with dense connections within each of the DFEMs (**bottom**).

Pedestrians in a crowded scene usually suffer from occlusion, high density, and perspective distortion. To address these challenges for enhancing counting accuracy, the estimated feature map must comprise low to complex, dense, and spatial-aware features. The general features with more spatial information extracted by the backbone network need to be propagated to higher layers without dissemination. Therefore, we directly appended the DFEM to the backbone network to propagate the general features to subsequent layers. Figure 3 (top) depicts the abstract view of the DFEM, whereas the detailed architecture is shown in Figure 3 (bottom). The DFEM consists of four DSCMs densely connected with each other as shown in Figure 3 (top). The upper DSCM accepts output from the lower ones, which results in aggregation of information from lower and lower-middle layers to upper layers. Further, task-independent general features extracted at lower layers are propagated to higher layers, thus combined with task-specific features extracted at higher layers. Further, DSCMs consist of three groups of convolution layers with different channel sizes as shown in Figure 3 (top). The dense connections among these groups enhance the ability of DSCMs to obtain and propagate not only dense features but also the spatial-aware features extracted at the lower layers.

#### Dense Stacked Convolution Module (DSCM)

The DFEM module consists of multiple DSCM modules densely connected with each other. Each DSCM follows the same dense connection pattern of a DFEM. In this way it is a dense network within a dense network. In our case, four DSCMs are densely connected with each other as shown in Figure 3 (top), whereas the internal architecture of the DSCM consists of three groups. In the first group, we have three convolution layers of the same size equal to 3×3, with different channel sizes like 256, 128, and 64. The second and third groups follow the same pattern. The output of the first group is passed to the second, third, and final layers of filter size 3×3 with 512 channels. In addition, the output of the second group is passed to the third group to obtain the intermediate density map. The dense nature of this module is very helpful for passing on the spatial as well as the general features from lower to higher layers. In addition, the multiple channels with different sizes are useful for learning deep features. In this way, a dense network within a dense network is very useful for combining the spatial features with task-specific features. Further, the information loss at the lower layers is minimized by propagating the learnable features to middle and higher layers.

### 3.3. Task-Independent and Task-Specific Feature Acquisition

The features extracted at lower and middle-lower layers to higher layers play a significant role in acquiring high segmentation accuracy [27]. Higher-layer features always contain more semantic information, and low-layer features contain more detailed information. A combination of the features extracted from low-level layers and high-level layers plays an important role for obtaining relevant contextual information. The backbone network is composed of the same size of filters with strong feature learning and transformation properties. The features extracted by the backbone network are disseminated while traveling to higher-level modules. To avoid this dissemination, dense connections among multiple DSCMs and within multiple convolutional groups of DSCMs are used to propagate the task-independent and -specific features to higher level layers. In this way, an effective combination of backbone network and DFEM plays a vital role in increasing the counting accuracy by aggregating detailed and semantic features. Especially, the DFEM with a densely oriented structure is useful for extracting and propagating the features to subsequent layers in a dense fashion. In other words, local information at each layer is propagated with the aggregation of global information in the final feature map.

### 3.4. Channel Attention Module (CAM)

A combination of low- and high-level features plays a vital role in enhancing the counting accuracy. DFEM is responsible for extracting and propagating the low- to high-level features. Class-specific features also play a significant role in enhancing the counting accuracy. We therefore incorporated the channel attention module (CAM) at the end to exploit the inter-dependencies among classes. The rich semantic feature representation at the final layers is obtained by modeling the dependency among channels, thus combining the low to high semantic features for enhancing counting accuracy. There are two types of class-specific responses in the CC domain: foreground (people region) and background (other region). Due to high density, multi-scaling, and perspective distortion, the foreground and background regions are occluded. Thus, incorporating the CAM is an effective way to reduce the estimation errors.

The architecture of CAM is shown in Figure 1 (bottom). Let us have an original feature map denoted by *X*. Firstly, input feature $X\in R^{H\times W\times C}$ is reshaped into $B\in R^{N\times C}$, whereas N=H×W. Secondly, reshaped *B* and the transpose of *X* are multiplied (matrix multiplication). Thirdly, softmax is applied to the output acquired in the previous step to obtain the attention map $A^{C\times C}$. The channel’s inter-dependencies are calculated by using $a_{ji}=\frac{exp(X_{i}X_{j})}{\sum_{i=1}^{C}exp(X_{i}X_{j})}$, such that $a_{ji}$ measures the $i_{th}$ channel’s impact on the $j_{th}$ channel. Further, we multiply the $X^{N\times C}$ by $A^{C\times C}$ and reshape the result to $R^{H\times W\times C}$. Lastly, the result is multiplied by a learnable value α and an element-wise summation is performed with the original *X* to obtain the output $Y\in R^{H\times W\times C}$. The final feature map is calculated by using $Y_{j}=\alpha \sum_{i=1}^{C}\left(a_{ji}X_{i}\right)+X_{j}$, which is a weighted sum of features of all channels and an original feature. This results in modeling of semantic inter-dependencies among channels. Therefore, CAM is applied to the final layers of the proposed HDPF-Crowd. Finally, point-wise prediction is obtained by applying softmax to the last feature map.

## 4. Implementation Details

The experimental detail of HADF-Crowd starts with network configuration to data preparation. The complete network architecture is shown in Table 1. Moreover, this section is further sub-divided into three sub-sections: network configuration, training details, and data preparation.

### 4.1. Network Configuration

The network configuration of the HADF-Crowd is shown in Table 1. The proposed approach is composed of three modules: backbone network, DFEM, and CAM. The backbone network comprises four sub-modules (Sub-M): Sub-M1 to Sub-M4. DFEM consists of Sub-M5 to Sub-M8. For the backbone network, we used the modified form of the VGG-16 network [26] with ten layers to minimize the computational complexity with the same and smaller sizes of filters [26]. The DFEM and four sub-modules (DSCMs) are densely connected with each other to obtain the dense features utilized in the higher layers. Further, CAM is placed at the end of the network to obtain the class-specific response between the background and foreground regions, thus combining the dense and high-level features for high CC accuracy.

### 4.2. Training Details

The loss between the estimated and ground truth density is calculated through Euclidean distance as given in Equation (1).

(1) $$L\left(\Theta \right)=\frac{1}{N}\sum_{i=1}^{N}{∥Z\left(X_{i},\Theta \right)-G_{i}∥}_{2}^{2}$$

where *N* denotes the total number of training images. The set of parameters is depicted by Θ, whereas $X_{i}$ is the input image and $G_{i}$ is its corresponding ground truth density map. Further, Z() is the model applied on the input image $X_{i}$ by optimizing the parameters to obtain the estimated density map. Moreover, the parameters are optimized by using stochastic gradient descent (SGD) with learning rate 1e-6 and momentum 0.9. For simulation, we used the PyTorch platform [28] with an NVIDIA GeForce GTX 1070.

### 4.3. Data Preparation

Data augmentation is performed by cropping the images in such a way that a total of 9 patches are cropped from a whole image. The size of each patch is 1/4 of the original image. The five patches are cropped randomly, and the rest of the four patches are the four corners of the input image. The mirroring is performed on the cropped image patches to enhance the training data. However, data augmentation is not performed for the test dataset.

## 5. Performance Evaluation

This section begins with evaluation metrics followed by test data used for evaluation of the proposed techniques. The purpose of this section is to evaluate the proposed approach on three well-known datasets: ShanghaiTech (Part-A), ShanghaiTech (Part-B), and Venice.

### 5.1. Metrics Used for Evaluation

To evaluate the proposed algorithm, we applied the most commonly used performance evaluation metrics; namely, mean absolute error (MAE) and mean square error (MSE) as given in Equations (2) and (3).

(2) $$MAE=\frac{1}{N}\sum_{i=1}^{N}\left|y_{i}-y_{i}^{{}^{\prime }}\right|$$

(3) $$MSE=\sqrt{\frac{1}{N}\sum_{i=1}^{N}{\left(y_{i}-y_{i}^{{}^{\prime }}\right)}^{2}}$$

where $y_{i}$ is the ground truth count and $y_{i}^{{}^{\prime }}$ is the estimated count for the *i*th training/testing sample. Further, *N* denotes the total number of training/testing samples.

### 5.2. Testing Data

#### 5.2.1. Venice Dataset

**Figure 4:**
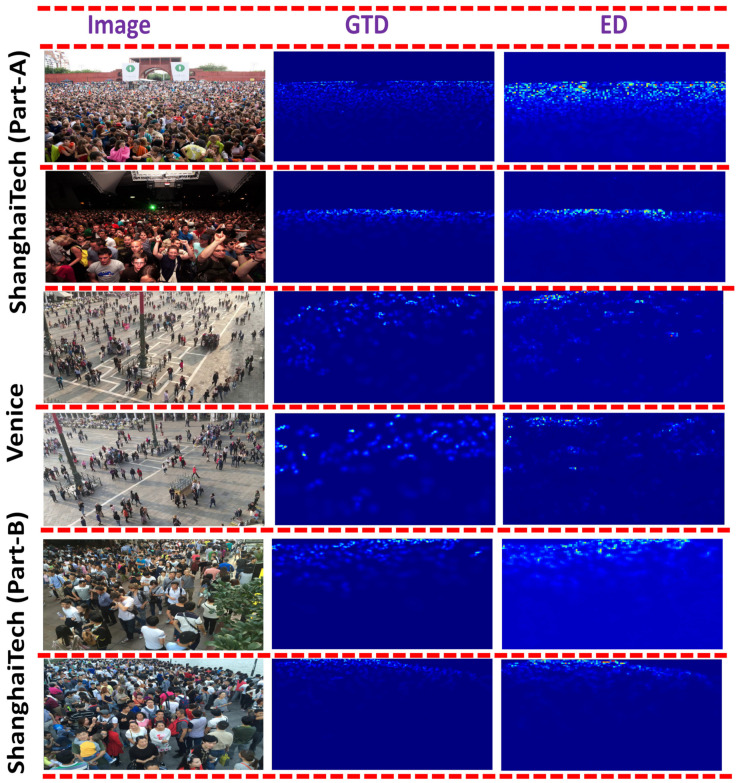
Visualization of ShanghaiTech (Part-A), (Part-B), and Venice datasets; ground truth density; estimated density.

Table 2 depicts the performance comparison between the proposed HADF-Crowd and the state-of-the-art techniques based on the MAE and MSE. It is observed that the HADF-Crowd provides the state-of-the-art performance as compared to rest of the techniques on the Venice dataset. The reason for the lowest error is due to strong transfer learning ability with extraction of low to complex features. Further, extraction and propagation of dense features to high-level layers increases the ability to obtain the scale-varying information. The CAM module with strong class-specific response further classifies the foreground and background regions, thus reducing the perspective distortion. The qualitative results are shown in Figure 4.

#### 5.2.2. ShanghaiiTech (Part-A)

The performance comparison between HADF-Crowd and the state-of-the-art techniques is shown in Table 2. A comparable performance is observed for the ShanghaiTech (Part-A) dataset. The reason is extraction of low to complex, deeper, and relevant features from lower, middle, and higher layers, providing better information. Dense connections among DSCMs are useful for propagating the information to higher layers. However, the CC accuracy of HADF-Crowd on ShanghaiTech (Part-A) is comparable due to the in-effectiveness of CAM modules in highly occluded environments. As the accuracy of CAM is more effective in low-density scenes, thus enabling classification of the foreground and background classes. However, the tilted behavior of ShanghaiTech (Part-A) towards high-density reduces the effectiveness of CAM, which results in high error rates. The qualitative results are shown in Figure 4.

#### 5.2.3. ShanghaiTech (Part-B)

A comparable performance has been shown in Table 2 for the ShanghaiTech (Part-B) dataset. The reason for this is the consideration of multi-variant features from lower- to high-level layers. Further, aggregation of task-independent and task-specific features extracted from lower and lower-middle layers enhances the counting accuracy. The combination of local and global features further enhances the counting accuracy by incorporating salient features for the final density map. However, ref. [38] has a low error rate for the ShanghaiTech (Part-B) dataset. This is due to consideration of pyramid pooling and nested dilated convolution, thus incorporating the contextual information with enhanced spatial sampling. The qualitative results are shown in Figure 4.

### 5.3. Ablation Study

This subsection is dedicated to investigating the capability of each component of HADF-Crowd. We conducted all ablations on the Venice dataset. To validate the effectiveness of HADF-Crowd, we conducted experiments by adding components incrementally as shown in Table 3. The ablation study consisted of three modules that were added sequentially.

- Backbone: Backbone is a VGG-16 based network.
- Backbone + DFEM: This consists of two modules. The sequential concatenation of Backbone with DFEM.
- Backbone + DFEM + CAM: Three modules are concatenated sequentially.

## 6. Conclusions and Future Work

In this work, we proposed a novel architecture called a hierarchical attention-based dense feature extraction through CNN for single image CC that is trained in an end-to-end manner. The general and spatial-aware features that are extracted by the backbone network are passed on to the next level (DFEM). Then, the DFEM with dense architecture is responsible for extracting and propagating the spatial-aware and dense features to the subsequent module CAM. CAM has the capability of strong class-specific responses, utilizing the features from lower modules to enhance the classification between background and foreground regions. Due to strong relevant feature aggregation properties from lower and lower-middle layers to higher layers, the performance of the network is enhanced in terms of counting accuracy. The combination of local and global features has been shown to be useful for improving the CC accuracy. The effective combination of general, deep, spatial-aware, and semantic features at the higher layers plays a vital role in high CC accuracy. More specifically, perspective distortion is countered by incorporating the CAM as a final module. In future, we intend to further investigate the CC domain by exploring the semantic segmentation.

## Figures and Tables

**Table 1:** The architecture of HADF-Crowd.

Modules	Sub-Modules	Channels	Filter	Padding	Dilation	HADF-Crowd
**Backbone Network**	Sub-M1	64	3 × 3	1	1	Conv3-64 Conv3-64 Max pooling
	Sub-M2	128	3 × 3	1	1	Conv3-128 Conv3-128 Max pooling
	Sub-M3	256	3 × 3	1	1	Conv3-256 Conv3-256 Max pooling
	Sub-M4	512	3 × 3	1	1	Conv3-512 Conv3-512 Conv3-512
**DFEM**	Sub-M5	512, 256, 128, 64	3 × 3	1	1	Conv3-512-1 Conv3-256-1 Conv3-128-1 Conv3-64-1
	Sub-M6	576, 256, 128, 64	3 × 3	1	1	Conv3-576-2 Conv3-256-2 Conv3-128-2 Conv3-64-2
	Sub-M7	640, 256, 128, 64	3 × 3	1	1	Conv3-640-2 Conv3-256-2 Conv3-128-2 Conv3-64-2
	Sub-M8	640, 512	3 × 3	1	1	Conv3-640-3 onv3-512-3
	Output	512, 128, 64, 1	3 × 3	1	1	Conv3-512-1 Conv3-128-1 Conv3-64-1 Conv1-1-1

**Table 2:** Estimation errors for ShanghaiTech (Part-A), (Part-B), and Venice datasets.

Technique	Part-A		Part-B		Venice	
	MAE	MSE	MAE	MSE	MAE	MSE
Marsden et al. [29]	126.5	173.5	23.8	33.1	-	-
MCNN [4]	110.2	173.2	26.4	41.3	145.4	147.3
C-MTL [30]	101.3	152.4	20.0	31.1	-	-
SwitchCNN [5]	90.4	135.0	21.6	33.4	52.8	59.5
SaCNN [31]	86.8	139.2	16.2	25.8	-	-
Mult-S-CNN [32]	83.7	124.5	17.9	32.4	-	-
CP-CNN [33]	73.6	106.4	20.1	30.1	-	-
ACSCP [34]	75.7	102.7	17.2	27.4	-	-
Deep-NCL [35]	73.5	112.3	18.7	26.0	-	-
IG-CNN [36]	72.5	118.2	13.6	21.1	-	-
CLPNet [37]	71.5	108.7	12.2	20.0	-	-
SCNet [38]	71.9	117.9	9.3	14.4	-	-
ic-CNN [39]	68.5	116.2	10.7	12.2	-	-
CSRNet [6]	68.2	115.0	10.0	16.0	35.8	50.0
DecideNet [40]	-	-	-	-	21.5	31.9
DRASAN [12]	69.3	96.4	11.1	18.2	-	-
DFE-Crowd [41]	71.6	110.9	9.7	16.0	23.8	34.5
IA-DCCN [42]	66.9	108.4	10.2	16.0	-	-
DsNet [41]	61.2	102.6	6.7	10.5	-	-
RANet [43]	59.4	102.0	7.9	12.9	-	-
ECAN [44]	62.3	100.0	7.8	12.2	20.5	29.9
HADF-Crowd	71.1	111.6	9.7	15.7	14.1	20.1

**Table 3:** An ablation study on the Venice dataset.

Modules	Venice Dataset	
	MAE	MSE
Backbone	43.0	60.2
Backbone + DFEM	23.8	34.5
Backbone + DFEM + CAM	14.1	20.1

## Data Availability

Not applicable.
